# Central aortic pressure and long-term outcome in hypertensive patients undergoing percutaneous coronary intervention

**DOI:** 10.1038/s41598-020-74619-3

**Published:** 2020-10-15

**Authors:** Han-Ping Wu, Mao-Jen Lin

**Affiliations:** 1 Department of Pediatric Emergency Medicine, China Medical University Children’s Hospital, China Medical University, Taichung, Taiwan; 2 Department of Medicine, College of Medicine, China Medical University, Taichung, Taiwan; 3 Department of Medical Research, China Medical University Children’s Hospital, China Medical University, Taichung, Taiwan; 4 Division of Cardiology, Department of Medicine, Buddhist Taichung Tzu Chi Hospital, The Buddhist Tzu Chi Medical foundation, 88, Section 1, Fong-Sing Rd, Tanzi District, Taichung, Taiwan; 5 Department of Medicine, College of Medicine, Tzu Chi University, Hualien, Taiwan

**Keywords:** Interventional cardiology, Cardiology

## Abstract

Elevated central pulse pressure (CPP) had a negative influence on long-term outcome in patients with hypertension (HT). However, little is known about the impact of central pulse pressure on long-term outcomes in hypertensive patients undergoing PCI. A total number of 1184 hypertensive patients who received PCI procedure were prospectively collected. They were divided into two groups according to the median of central pulse pressure. Baseline characteristics, risk factors, hemodynamic data including central systolic pressure (CSP), central diastolic pressure (CDP) and CPP were measured. Invasive strategies were also analyzed to compare the long term outcome between patients with reference CPP and patients with high CPP. We further analyzed the predictors for myocardial infarction (MI), mortality, repeated PCI procedure in hypertensive patients undergoing PCI. We found patients in the reference CPP group had a lower CSP and higher CDP compared with high CPP group (Both P < 0.001) and male preponderance (P < 0.001). Patients with diabetes and chronic kidney disease (CKD) tend to have a high CPP (both P < 0.001). Drugs including Angiotensin Converting Enzyme inhibitors (ACEI) and statin were used more frequently in patients with reference CPP group (P = 0.035 and P = 0.001, respectively). Freedom from all-cause mortality and cardiovascular(CV) mortality was lower in the patients with high CPP group (P = 0.001, P = 0.01, respectively).Logistic regression revealed that CPP is a major predictor for all-cause mortality and repeated PCI procedure [hazard ratio (HR): 2.46 and 1.41, respectively]. In hypertensive patients receiving PCI, elevated CPP had a negative impact on long-term mortality; CPP also strongly predicts all-cause mortality and repeated PCI procedures in hypertensive patients undergoing PCI.

## Introduction

Percutaneous coronary intervention (PCI) is a common therapeutic strategy in patients with coronary artery disease (CAD). Atherosclerotic plaque can be treated via different techniques such as expansion of the lumen by stretching and tearing the plaque (eg, balloon angioplasty), scaffolding the plaque (eg, coronary stents), removing the plaque (eg, atherectomy), or ablating the plaque (eg, laser angioplasty). However, even with introduction of modern techniques, adverse events still occurred in a high proportion of patients after receiving PCI. Major adverse cardiovascular event (MACE) of patients undergoing PCI include myocardial infarction (MI), revascularization and death^1^. Major risk factors for developing CAD including diabetes mellitus (DM), hypertension, dyslipidemia, and chronic kidney disease could also affect outcomes in CAD patients receiving PCI^2–8^.

Central pulse pressure (CPP) is equal to central systolic pressure minus central diastolic pressure. Recently, CPP has been proved to strongly associated with cardiovascular outcome and might serve as a better predictor than brachial pressure^9–12^ in patients with hypertension. For patients undergoing repeated PCI, CPP was also strongly related with MI attack, CV mortality, and all-cause mortality^13^. Nevertheless, there is no study thus far as for central aortic pressure and long-term outcome focusing on hypertensive patients undergoing PCI. Therefore, in this study, we aimed to evaluate the role of CPP in hypertensive patients receiving PCI, and compare the long term outcome between patients with reference CPP or high CPP value. We further analyzed the major predictors for myocardial infarction (MI), repeated PCI and mortality in hypertensive patients undergoing PCI.

## Methods and materials

### Study population

The study was a clinical cohort based on prospective design and conducted via medical record and catheterization data review from 2007 through 2018. We consecutively recruited hypertensive patients undergoing PCI patients aged between 20 and 90 years from the inpatient clinic at Taichung Tzu Chi Hospital, Taiwan. According to median of CPP, they were divided into two groups: hypertensive patients with reference CPP (CPP equal to or less than median); hypertensive patients with high CPP (CPP more than median). Patients with the following conditions: scheduled PCI, end-stage heart failure, underlying malignancy, and occurrence of major adverse cardiovascular event (MACE) within 30 days after undergoing PCI were all excluded. The primary end-points include myocardial infarction, cardiovascular mortality, all-cause mortality and repeated PCI. Most patients were followed up regularly via the outpatient department (OPD) basis. For very few patients lost follow-up at OPD, usually a telephone call would be used to contact the patients themselves or their families. The Institution Review Board and ethics committee of Taichung Tzu Chi Hospital approved the study protocol. This cohort study also fulfilled the guidance of Strengthening the Reporting of Observational Studies in Epidemiology (STROBE) statement^14^.

### Data processing, measurements and analysis

Comparisons of baseline characteristics, hemodynamic data on cardiac catheterization, major risk factors and differences between treatment strategies such as drug medications after PCI or invasive procedures (balloon angioplasty, bare metal stent deployment or drug-eluting stent deployment) were all gathered in our study. The measurements of body habitus included body height, body weight, and body mass index (BMI). Baseline biochemical data including fasting plasma glucose, creatinine, total cholesterol (TC), high-density lipoprotein-cholesterol (HDL-C), low-density lipoprotein-cholesterol (LDL-C), serum triglyceride and creatinine level during index PCI were all collected. HT is defined as a usual BP of 140/90 mm Hg or higher, BP levels for which the benefits of pharmacologic treatment have been definitely established^15^. Diabetes was defined as a fasting plasma glucose level of more than 126 mg/dL, a casual plasma glucose level greater than 200 mg/dL or a hemoglobin A1c (HbA1c) level of more than 6.5%^16^. Hypercholesterolemia was defined as a serum cholesterol level of more than 200 mg/dL or an LDL-C level of more than 100 mg/dL. Chronic kidney disease (CKD) was defined as an estimated glomerular filtration rate (eGFR) of less than 60 ml/min/1.73 m^2^, which is equal to or more than stage III chronic kidney disease (CKD)^17^. Previous MI history was defined as a history of MI prior to first-time PCI, accompanied by a threefold elevation of cardiac enzymes from the baseline value.

Before coronary angiography, a cocktail including nitroglycerine 300 µg, verapamil 5 mg and heparin 5000 units were injected into radial artery after successful puncture. The central aortic pressure (CAP) was measured via a pigtail catheter while performing a coronary angiography. As for the hemodynamic data, CAP including CSP, CDP, and CPP were measured continuously and mean value were calculated during the whole time of catheterization. Angiographic findings, including the number of diseased vessels and lesion locations were calculated and lesion severity and complexity were evaluated via the synergy between PCI with Taxus Express paclitaxel-eluting stent (Boston Scientific, Marlborough, MA, USA) and cardiac surgery score (SYNTAX score)^18^. Related clinical parameters including baseline characteristics, hemodynamic data, related risk factors and invasive strategies were compared between patients with reference group and high CPP group. In addition, we intended to identify the significant predictors for major adverse outcomes including myocardial infarction (MI), all-cause mortality, CV mortality and repeated PCI procedures.

### Statistical analysis

The analysis was primarily used to compare the differences between the two groups. Analysis of variance (ANOVA) was used to test continuous variables. Pearson’s chi-squared test, Fisher’s exact test was used to examine categorical variables. The log-rank test and Kaplan–Meier curves were used for the survival analysis. The Cox proportional hazards model was used to test the effect of independent variables on hazards. *P* values less than 0.05 were considered significant. All analyses were performed by using the statistical package SPSS for Windows, Version 23.0 (IBM Corp., Armonk, NY, USA).

### Ethics approval and consent to participate

The study protocol was approved by the Institution Review Board and ethics committee of Taichung Tzu Chi Hospital, Taiwan (REC108-12), and written informed consent was obtained from all study participants.

## Results

During the study period, a total of 1184 hypertensive patients who underwent PCI procedure were collected. The median of CPP according to cardiac catheterization is 66 mmHg. Based on the median of CPP, 1184 patients were divided into two groups. 601 patients and 583 patients were classified into reference CPP and high CPP group, respectively. The mean follow-up time for low CPP and high CPP group was 227.5 ± 135 weeks versus and 229.2 ± 133.0 weeks, respectively (P = 0.823).

The baseline clinical characteristics were listed in Table 1. Patients with high CPP group were older than that with reference CPP group (69.2 ± 10.8 vs. 62.4 ± 12.4 years old, P < 0.001). They also had a higher serum creatinine level (2.3 ± 2.7 mg vs 1.6 ± 2.0 mg, P < 0.001). As for the body habitus parameters, patients in the high CPP group had a lower body mass index (BMI) than patients in the reference CPP group (P = 0.003). Given the hemodynamic data, patients with high CPP group had a higher CSP (158.7 ± 19.0 vs. 127.2 ± 16.4 mmHg) and a lower CDP (72.9 ± 12.4 vs. 75.7 ± 14.0 mmHg) than those with reference CPP group (P < 0.001; P = 0.006, respectively).

**Table 1:** General characteristic of study population.

Variable	Reference CPP (N = 601)	High CPP (N = 583)	P value
Age (years)	62.4 ± 12.4	69.2 ± 10.8	< 0.001*
Weight (kg)	71.3 ± 13.3	65.7 ± 12.2	< 0.001*
Height (m)	1.64 ± 0.08	1.60 ± 0.08	< 0.001*
BMI (kg/m^2^)	26.4 ± 4.3	25.7 ± 4.3	0.003*
CSP (mmHg)	127.2 ± 16.4	158.7 ± 19.0	< 0.001*
CDP (mmHg)	75.7 ± 14.0	72.9 ± 12.4	< 0.001*
CPP (mmHg)	51.5 ± 9.7	85.8 ± 16.0	< 0.001*
Cholesterol (mg/dL)	175.8 ± 44.2	176.3 ± 43.7	0.844
HDL (mg/dL)	38.2 ± 15.3	39.6 ± 17.2	0.141
TG (mg/dL)	156.0 ± 106.4	158.7 ± 104.0	0.666
LDL (mg/dL)	106.3 ± 38.2	104.9 ± 37.5	0.536
Serum creatinine (mg/dL)	1.6 ± 2.0	2.3 ± 2.7	< 0.001*

BMI: body mass index, CSP: central systolic pressure, CDP: central diastolic pressure, CPP: central pulse pressure, HDL: high-density lipoprotein cholesterol, LDL: low-density lipoprotein cholesterol, TG: triglyceride.

*Significant.

The demographic data of the study population is shown in Table 2. Female was more prone to have high CPP than male (P < 0.001). Patients with DM or CKD also have a higher CPP level than those without DM or CKD. In addition, we found patients in the reference group used more ACEI inhibitors and statin but less calcium channel blockers

**Table 2:** Demography and clinical data of study population and medications prescribed after index PCI.

Variable	Reference CPP (%) (N = 601)	High CPP (%) (N = 583)	P value
**Gender**			< 0.001*
Female	110 (18.3)	238 (40.8)	
Male	491 (81.7)	345 (59.2)	
**Dyslipidemia**			0.460
No	284 (47.3)	288 (49.4)	
Yes	317 (52.7)	295 (50.6)	
**DM history**			< 0.001*
No	363 (60.4)	277 (47.5)	
Yes	238 (39.6)	306 (52.5)	
**Current smoker**			< 0.001*
No	330 (54.9)	405 (69.5)	
Yes	271 (45.1)	178 (30.5)	
**CKD**			< 0.001*
No	367 (61.1)	226 (38.8)	
Yes	234 (38.9)	357 (61.2)	
**Previous MI**			< 0.001*
No	383 (63.7)	440 (75.5)	
Yes	218 (36.3)	143 (24.5)	
**Stroke history**			0.163
No	565 (94.0)	536 (91.9)	
Yes	36 (6.0)	47 (8.1)	
**CABG history**			1.000
No	596 (99.2)	579 (99.3)	
Yes	5 (0.8)	4 (0.7)	
**Aspirin**			0.905
No	60 (10.0)	57 (9.8)	
Yes	541 (90.0)	526 (90.2)	
**P2Y12 inhibitor**			0.171
No	79 (13.1)	93 (16.0)	
Yes	522 (86.9)	490 (84.0)	
**Diuretics**			0.516
No	468 (77.9)	463 (79.4)	
Yes	133 (22.1)	120 (20.6)	
**BB**			0.347
No	298 (49.6)	305 (52.3)	
Yes	303 (50.4)	278 (47.7)	
**CCB**			0.001*
No	398 (66.2)	330 (56.6)	
Yes	203 (33.8)	253 (43.4)	
**ACEI**			0.035*
No	495 (82.4)	506 (86.8)	
Yes	106 (17.6)	77 (13.2)	
**ARB**			0.084
No	386 (64.2)	346 (59.3)	
Yes	215 (35.8)	237 (40.7)	
**Statin**			0.001*
No	320 (53.2)	368 (63.1)	
Yes	281 (46.8)	215 (36.9)	
**Fibrate**			0.730
No	562 (93.5)	548 (94.0)	
Yes	39 (6.5)	35 (6.0)	

Previous MI: history of previous myocardial infarction, CABG: *history* history of coronary artery bypass graft, CKD: chronic kidney disease, P2Y12 inhibitor: P2Y12 receptor inhibitor of platelet, BB: beta-blockers, CCB: calcium channel blocker, ACEI: angiotensin-converting enzyme inhibitor, ARB: angiotensin receptor blocker.

P value for Pearson's chi-squared test or Fisher’s exact test.

**Figure 1:**
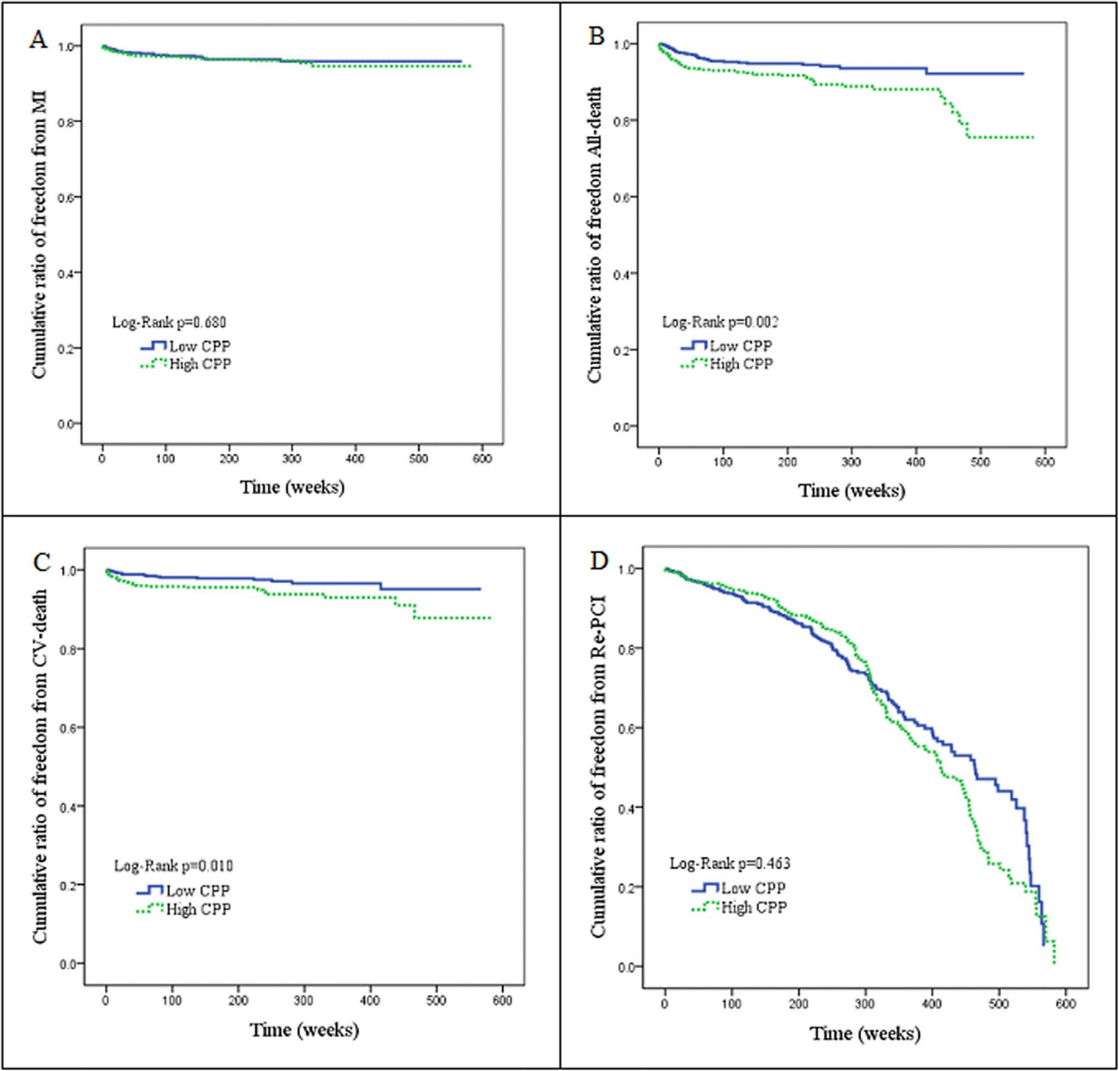
**(A)** Cumulative ratio of freedom from MI between two groups (P = 0.680). **(B)** Cumulative ratio of freedom from All-death between two groups (P < 0.002). **(C)** Cumulative ratio of freedom from CV-death between two groups (P < 0.010). **(D)** Cumulative ratio of freedom from Re-PCI between two groups (P < 0.463).

*Significant.

(CCB) compared to those in the with high CPP group (P = 0.035, P = 0.001, P = 0.001, respectively). The results of angiographic findings and clinical outcomes are shown in Table 3. There is no difference as for the distribution of diseased vessels between both group; however, patients with high CPP tend to have more treated lesions (P = 0.044). They also received more balloon angioplasty (P = 0.013) but fewer drug eluting stent (DES) deployment (P = 0.046). Patients with high CPP group had a higher rate of all-cause mortality and CV mortality than reference CPP group (P = 0.002, P = 0.01, respectively), but there was no difference int terms of MI or repeated PCI procedure (P = 0.678, P = 0.399, respectively). Figure 1 revealed the cumulated rate of freedom from MI, CV death, all-cause death and repeated PCI procedures between the two groups. Freedom from all-cause death and CV death was lower in the high CPP group (P = 0.002, P = 0.01, respectively).

**Table 3:** Demography of angiographic findings and clinical outcome.

Variable	Reference CPP (%) (N = 601)	High CPP (%) (N = 583)	P value
Follow-up time (weeks)	227.5 ± 135.0	229.2 ± 133.0	0.823
**Number of diseased vessels**			0.243
Single-vessel disease	279 (46.4)	249 (42.7)	
Dual-vessel disease	189 (31.4)	182 (31.2)	
Triple-vessel disease	133 (22.1)	152 (26.1)	
Mean of treated vessels	1.2 ± 0.5	1.3 ± 0.5	0.100
Mean of treated lesions	1.5 ± 0.7	1.6 ± 0.8	0.044*
**Type of intervention**			
Balloon angioplasty	162 (27.0)	196 (33.6)	0.013*
BMS deployment	239 (39.8)	258 (44.3)	0.118
DES deployment	280 (46.6)	238 (40.8)	0.046*
SYNTAX score	11.3 ± 8.1	10.3 ± 6.9	0.023*
LVEF	0.5 ± 0.1	0.6 ± 0.1	0.014*
**MI**			0.678
Yes	20 (3.3)	22 (3.8)	
No	581 (96.7)	561 (96.2)	
**CV death**			0.010*
Yes	16 (2.7)	33 (5.7)	
No	585 (97.3)	550 (94.3)	
**All-cause death**			0.002*
Yes	33 (5.5)	60 (10.3)	
No	568 (94.5)	523 (89.7)	
**Re-PCI**			0.399
Yes	158 (26.3)	166 (28.5)	
No	443 (73.7)	417 (71.5)	

BMS: bare metal stent, DES: drug-eluting stent, SYNTAX score: Synergy between Percutaneous Coronary Intervention with Taxus and Cardiac Surgery score, LVEF: left ventricular ejection fraction, MI: myocardial infarction, Re-PCI: repeated percutaneous coronary intervention.

*Significant.

The related factors in predicting the MACE are shown in Table 4. Multivariate regression was used for analysis. Based on the results of Cox regression model, we found that high CPP, advanced age, previous MI would increase the risk of all-cause mortality, whereas usage of statins and preserved left ventricular ejection fraction (LVEF) would reduce the risk. Advanced age and previous MI would increase the risk of CV mortality, however; usage of CCB and preserved LVEF would reduce the risk, Finally, high CPP, male gender, presence of CKD and more treated lesions during index PCI would increase the risk of repeated PCI procedures; on the other hand, usage of statins and preserved LVEF would reduce the risk of repeated PCI procedures.

**Table 4:** Significant predictors of outcome in Cox proportion hazard model for MI, all-death, CV-death, repeated PCI by using multivariate regression.

Variables	MI^a^	All-death^b^	CV-death^c^	Repeated PCI^d^
	HR (95% CI)	HR (95% CI)	HR (95% CI)	HR (95% CI)
Reference CPP	1.00	1.00	1.00	1.00
High CPP	1.08 (0.48–2.42)	2.46 (1.26–4.77)*	2.09 (0.86–5.05)	1.41 (1.02–1.95)*
Age	1.04 (0.99–1.07)	1.04 (1.01–1.07)*	1.05 (1.01–1.09)*	1.00 (0.98–1.01)
BMI	1.06 (0.96–1.17)	1.02 (0.94–1.10)	1.05 (0.95–1.16)	1.00 (0.96–1.04)
Male gender	1.64 (0.67–4.03)	1.46 (0.75–2.83)	0.66 (0.28–1.58)	1.49 (1.02–2.18)*
DM	1.76 (0.81–3.81)	0.98 (0.54–1.78)	1.10 (0.49–2.46)	1.14 (0.85–1.53)
Smoking	0.50 (0.20–1.29)	0.73 (0.35–1.49)	0.97 (0.36–2.67)	1.28 (0.92–1.78)
CKD	0.94 (0.37–2.36)	1.26 (0.59–2.68)	0.99 (0.37–2.62)	1.51 (1.06–2.14)*
MI history	2.29 (0.99–5.26)	2.79 (1.48–5.26)*	2.75 (1.16–6.51)*	1.35 (0.97–1.90)
CCB	1.13 (0.50–2.57)	0.59 (0.30–1.16)	0.26 (0.09–0.79)*	1.04 (0.77–1.41)
ACEI	1.98 (0.86–4.55)	0.67 (0.32–1.43)	0.60 (0.21–1.71)	0.85 (0.60–1.20)
Statin	0.56 (0.23–1.41)	0.41 (0.19–0.85)*	0.49 (0.19–1.27)	0.72 (0.53–0.98)*
Treated lesions	1.20 (0.77–1.86)	0.86 (0.60–1.25)	0.80 (0.47–1.35)	1.42 (1.21–1.66)*
Balloon angioplasty	0.66 (0.29–1.54)	0.85 (0.44–1.68)	0.70 (0.28–1.76)	0.86 (0.61–1.21)
DES	0.43 (0.17–1.09)	1.08 (0.57–2.05)	0.71 (0.29–1.77)	1.11 (0.82–1.51)
SYNTAX score	0.99 (0.94–1.05)	1.04 (1.00–1.08)	1.02 (0.97–1.08)	0.99 (0.97–1.01)
LVEF	0.09 (0.01–1.40)	0.04 (0.01–0.31)*	0.02 (0.01–0.42)*	0.32 (0.11–0.98)*

CPP: central pulse pressure, BMI: body mass index, CKD: chronic kidney disease, MI history: previous history of myocardial infarction, BB: beta-blockers, CCB: calcium channel blocker, ACEI: angiotensin-converting enzyme inhibitor, DES: drug-eluting stent, SYNTAX score: Synergy between Percutaneous Coronary Intervention with Taxus and Cardiac Surgery score, LVEF: left ventricular ejection fraction.

*Significant.

^a^MI model: y = βdummyDH1 + βdummyDH2 + βdummyDH3 + βMI + βstroke + βstatin + βsyntax..

^b^All-death model: y = βdummyDH1 + βdummyDH2 + βdummyDH3 + βage + βCKD + βMI + βstroke + βbetab + βstatin + βsyntax..

^c^CV-death model: y = βdummyDH1 + βdummyDH2 + βdummyDH3 + βMI + βstroke + βdiuretics + βbetab + βACEI + βstatin + βsyntax..

^d^Repeated PCI model: y = βdummyDH1 + βdummyDH2 + βdummyDH3 + βMI + βsmoking + βbetab + βsyntax..

## Discussion

It has been well established that central pressure is a more accurate predictor for vascular disease and outcome than brachial pressure. However, the impact of central pressure on long-term outcomes in hypertensive patients with CAD undergoing PCI remains obscure. In the current study, we found that in hypertensive patients receiving PCI, high CPP had a negative impact on long-term all-cause mortality and CV mortality; high CPP also strongly predicts all-cause mortality and repeated PCI procedures in hypertensive patients undergoing PCI.

In our study, we found a high CPP was a strong factor for patients undergoing PCI procedure. As shown in methodology in CAFÉ study, the CPP was calculated via indirect measurement through radial artery applanation tonometry and pulse wave analysis was also recorded; whereas in our study the CPP was continuously measured and the mean value was calculated during the whole catheterization time via a pigtail catheter, this may have more exactly reflected the true central pressure and vessel wall stress than that in CAFÉ study. In current study, hypertensive patients with poor control group also had a higher CSP and a lower CDP than that in hypertensive patients with good control group; in other words, hypertensive patients with poor control might have more advanced arterial stiffness and atherosclerosis than those with good control. In another study for patients with end-stage renal disease, central pressure had a more predictive value for MACE than did brachial artery pressure^19^. Therefore, we think it is an important issue to control CPP tightly in order to achieve a better outcome in hypertensive patients undergoing PCI.

In hypertensive patients with high CPP, they have a preponderance for aged people, female patients, patient with CKD or diabetes. In aged people group, it also have a high percentage of isolated systolic hypertension (ISH), high CPP will be measured and might thus confound the result. These patients also have a poor control of blood pressure even though they need more strict blood pressure control. On the other hand, aged and female hypertensive patients, hypertensive patient with CKD or DM are also at high risk for advanced atherosclerosis, and they may have a poor outcome even after undergoing PCI^20^. In addition, calcium channel blockers (CCB) was used more frequently in the high CPP group, whereas ACEI and statins were used less frequently in high CPP group. It might imply that patients with higher CPP were older and had a high prevalence of ISH, therefore they used CCB more frequently. ACEI has been proved to effective in reducing cardiovascular event in stable CAD patients either with low or high risk^21,22^. It is unclear why patients with reference CPP used ACEI and statin more frequently. Nevertheless, ARB seemed have less protective effect than ACEI in patients with diabetes or acute myocardial infarction(AMI) undergoing PCI from post-hoc analysis^23^, whereas they also play a similar role in hypertensive patients is uncertain. On the other hand, both the serum TC or LDL-C value are in average level and there is no difference between two groups; however patients with reference CPP use statin more frequently and statin usage does reduce the hazard of all-cause mortality and repeated PCI from regression model. Currently, statin usage is recommended in all patient undergoing PCI even they have average cholesterol or LDL level.

The distribution of diseased vessels is not different between two groups. But drug eluting stent (DES) deployment was performed more frequently in patients with low CPP group whereas balloon angioplasty was used more frequently in patients with high CPP group. Paradoxically, SYNTAX score is higher in patients with reference CPP group but treated lesions were more in patients with high CPP group and only treated lesions increased hazards of repeated PCI procedure .This imply that treated lesions rather than SYNTAX score might actually reflect the extent of coronary atherosclerosis. Interventional clinicians should pay more attention to treated lesions during PCI procedure; once the number of treated lesion increases, the possibility of repeated PCI will also increase.

Furthermore, we analyzed the outcome differences between patients with high CPP and those with reference CPP. In this study, we found that hypertensive patients with high CPP has higher all-cause mortality and CV mortality than those with reference CPP .Based on the results of Cox proportional hazards mode, elevated CPP, age, previous history of MI will increase the hazards of all-cause mortality while elevated CPP, male gender, presence of CKD will increase the risk for repeated PCI procedure. CPP could be a very important predictor for clinical outcome in hypertensive patients receiving PCI. Carefully monitoring and controlling CPP by non-invasive method such as radial artery applanation tonometry and pulse wave analysis might be considered in hypertensive patients after undergoing PCI since invasive method to monitor CPP after cardiac catheterization is nearly impossible.

## Study limitation

First, the adherence to anti-hypertensive therapy was not surveyed in this study, which could have a influence on the long-term outcome. Second, data-entry bias might exits, functional evaluations of the atherosclerotic lesions by fraction flow reserve (FFR) or instantaneous wave-free ratio (IFR) measurement were not used in this study, which may also have an impact on the decision of index PCI. Third, the case numbers of MI and CV death are too few to yield statistical significance; we cannot exclude the possibility of inadequate follow-up time. Fourth, since most hypertensive patient receiving PCI in this study were aged people, this study may underpower the outcome of younger patients. Fifth, vasodilators and vasopressors had been used occasionally during PCI procedures in some conditions such as high or low blood pressure, coronary artery spasm and slow coronary flow, which might affect the mean CPP. Finally, since this is a observation study, whether aggressive lowering CPP could improved long-term outcome in hypertensive patients undergoing PCI remains obscure.

## Conclusions

Elevated CPP during PCI in hypertensive patients carried a increased long-term mortality, and CPP may also serve as a predictor for all-cause mortality and repeated PCI procedures in hypertensive patients undergoing PCI.

## Data Availability

The data that support the findings of this study are available, on reasonable request, from the corresponding author.
